# Thirty Minutes to Transform Care: A Mixed-Methods Study on Brief Psychosomatic Education for Unexplained Symptoms

**DOI:** 10.7759/cureus.84091

**Published:** 2025-05-14

**Authors:** Sasha R Sioni, Nathan Carroll, Jay Al-Hashimi, Lesley Manson

**Affiliations:** 1 Medical Education, Florida International University, Herbert Wertheim College of Medicine, Miami, USA; 2 College of Health Solutions, Arizona State University, Phoenix, USA; 3 Psychiatry, Jersey Shore University Medical Center, Neptune Township, USA; 4 Healthcare Leadership, Brown University, Providence, USA

**Keywords:** biopsychosocial approach, brief educational intervention, clinician empathy training, medically unexplained symptoms, mixed-methods research, patient-centered care, primary care communication, psychosomatic education

## Abstract

Introduction: Communicating effectively with patients who present medically unexplained physical symptoms (MUPS) remains a notable challenge for primary care clinicians. Despite the frequency of MUPS in primary care, few targeted educational interventions focus on improving communication skills for these encounters.

Methods: This single-group, pre-post study evaluated the impact of a concise, 30-minute psychosomatic training module at a large community health center in Phoenix, Arizona (United States). Eighty primary care clinicians (physicians, physician assistants, nurse practitioners, and nurses) received brief didactics on the biopsychosocial model, followed by a case-based discussion and role-play illustrating empathic validation techniques. Assessment measures included (1) the Adapted Somatic Symptom Scale-8 (for MUPS recognition), (2) the Psychosomatic Illness Knowledge Questionnaire, and (3) self-reported knowledge and comfort (KCTMQ). Qualitative reflections were also collected. Wilcoxon signed-rank tests, paired t-tests, and thematic analysis were used to examine changes and capture participant feedback.

Results: Significant pre-post improvements (p < 0.0001) were observed in MUPS recognition (Cohen’s d = 2.04), psychosomatic knowledge (d = 0.94), communication knowledge (d = 0.88), and comfort (d = 0.79). Qualitative data revealed intentions to integrate psychosocial factors earlier in clinical visits, employ validation statements more frequently, and convey increased confidence when addressing mind-body connections.

Conclusion: A short, 30-minute psychosomatic training session can substantially enhance clinicians’ communication competencies for MUPS. Even brief, well-structured interventions may help clinicians better recognize somatic symptoms, validate patient experiences, and apply biopsychosocial principles. Embedding such training into medical education and continuing professional development programs provides a feasible strategy to address communication gaps and potentially improve care for patients with unexplained symptoms.

## Introduction

Despite affecting nearly one in four primary-care consultations and generating billions of dollars in annual healthcare expenditures, medically unexplained physical symptoms (MUPS) remain a critical communication blind spot, where biomedical training fails to equip clinicians with essential psychosocial communication skills [1,2]. These persistent complaints, typically involving chronic pain, fatigue, or gastrointestinal disturbances, often prompt repeated diagnostic work-ups that fail to identify clear biomedical causes [2-4]. Within this clinical context, communication difficulties emerge as a central barrier to effective care. Patients frequently report feeling dismissed or misunderstood when providers cannot offer concrete explanations for their symptoms, while clinicians often experience frustration because they cannot provide satisfying answers [3]. This communication gap therefore represents a crucial yet underdeveloped area in medical-education research.

The biopsychosocial model provides a foundational framework for understanding MUPS, emphasizing the complex interplay among physiological mechanisms, psychological factors, and social determinants [5]. Despite compelling evidence supporting this integrated approach, postgraduate interventions that target communication about psychosomatic contributors have achieved only partial penetration of routine practice [6]. Recent scholarship in philosophical psychiatry further argues that fully realizing the biopsychosocial model requires explicit attention to external social contexts and meaning-making during clinical encounters [7]. Even so, the single-group pre/post design remains a pragmatic evaluation strategy in medical-education research, despite known methodological limitations [8]. Scoping and survey investigations of undergraduate curricula now confirm that instruction on psychosocial contributors to physical symptoms is taught sporadically and inconsistently [9,10]. This educational deficiency leaves clinicians ill-equipped to validate patient experiences while simultaneously exploring potential psychological and social dimensions of their complaints.

While previous studies have examined multifaceted interventions for MUPS management, a significant gap persists in evaluating brief, focused training modules that specifically address the communication challenges inherent in these encounters [6]. The present study addresses this gap by introducing and evaluating a 30-minute psychosomatic education intervention designed to enhance clinician-patient communication about unexplained symptoms. By focusing explicitly on communication strategies rather than diagnostic algorithms, this research contributes to the emerging literature on educational approaches for teaching MUPS management. The study examines how short-format education can affect clinicians’ recognition of psychosocial contributors, comfort discussing mind-body connections, and ability to provide validating responses that acknowledge symptom reality while avoiding unnecessary medicalization [1,3,4].

This investigation aims to determine whether a concise educational intervention can meaningfully improve communication competencies for MUPS management in healthcare professionals, potentially enhancing clinical practice without requiring extensive curricular restructuring. The findings have direct implications for continuing medical education, communication-skills training, and the development of focused educational approaches to medically unexplained symptoms across healthcare settings [2,7].

## Materials and methods

Study design and theoretical framework

This single-group, pre-post study evaluated a concise, 30-minute psychosomatic training module aimed at enhancing clinician-patient communication about MUPS. The initiative was framed as a quality-improvement project in response to unmet needs in clinician communication skills for MUPS [11-14]. While the lack of a control group limits causal inference, this approach offers pragmatic insight into the intervention’s potential impact [15,16].

The module drew on four key concepts: (1) biopsychosocial model-integration of biological, psychological, and social factors [17,18]; (2) adult-learning theory-brief, problem-centered activities that facilitate immediate skill application [19]; (3) clinician empathy-empathic engagement to foster patient trust and reduce unnecessary testing [20]; and (4) micro-learning-focused educational units that improve knowledge retention in health-professions training [21].

Operational definitions

Each construct was translated into an actionable teaching objective: (1) biopsychosocial model-participants practiced eliciting at least one psychological and one social contributor in addition to biomedical data, consistent with Engel’s framework [17]; (2) clinician empathy-role-play feedback required the clinician to demonstrate understanding of the patient’s perspective, communicate that understanding, and propose a mutually agreed action plan [20]; (3) micro-learning-content was packaged into ≤ 15-minute units with one measurable learning objective and an immediate knowledge check [21]; and (4) adult-learning theory activities were self-directed, problem-centered, and immediately applicable to participants’ own clinical cases [19].

Setting and participants

The study was conducted at a large community health center in Phoenix, Arizona (United States). Eligible participants were physicians, nurse practitioners, physician assistants, and nurses who (i) provided direct patient care, (ii) were proficient in English, and (iii) agreed to complete both pre- and post-intervention assessments. Participation was voluntary; no monetary or time-release incentives were provided. A priori power analysis (two-tailed, α = 0.05) indicated that a minimum of 78 paired observations would provide 95% power to detect a medium effect (Cohen's d = 0.45) on the primary communication-skill outcome. Therefore, the final sample of 80 met this precision criterion while remaining feasible for a quality-improvement study.

Recruitment and flow

**Figure 1:**
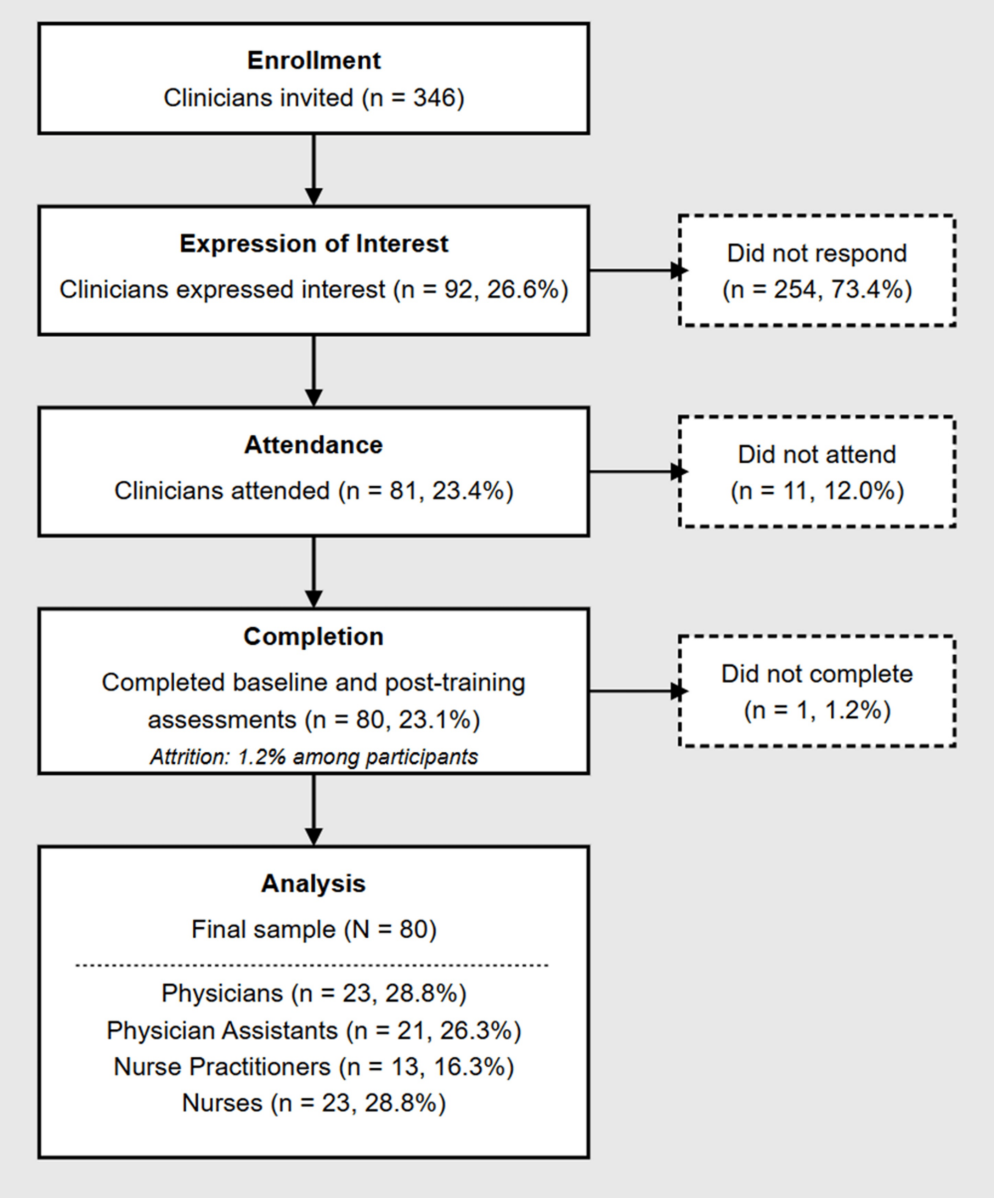
CONSORT flow diagram of clinician participation CONSORT‑style flow diagram outlining recruitment and retention of 346 clinicians invited, 92 (26.6%) expressed interest, 81 (23.4%) attended the 30‑minute session, and 80 (23.1%) completed pre‑ and post‑training surveys (1.2 % attrition). The analytic cohort included 23 physicians, 21 physician assistants, 13 nurse practitioners, and 23 nurses.

A total of 346 clinicians were invited to take part in the 30-minute training. Because participation occurred during routine clinic hours and no protected time or financial incentive was provided, most non-responders cited scheduling conflicts or workload pressures when declining the invitation; a smaller subset were on leave during the study window. Although these reasons were captured informally through opt-out e-mails and administrative logs, no systematic data collection was undertaken. Overall non‑response was 76.9% (266/346), reflecting the gap between 346 invitations and 80 complete data sets. Ninety-two clinicians (26.6 %) expressed interest, 81 (23.4 %) attended, and 80 (23.1 %) completed both baseline and immediate post-training assessments (Figure 1). One participant failed to submit post-training questionnaires, yielding a 1.2% attrition rate. Convenience sampling ensured feasibility but may have introduced selection bias favoring clinicians already motivated to improve MUPS communication [11,22].

Sample characteristics

The final sample (N = 80) comprised 23 physicians (28.8%), 21 physician assistants (26.3%), 13 nurse practitioners (16.3%), and 23 nurses (28.8%). Overall, 41 participants (51.3%) self-identified as female and 39 (48.8%) as male. The mean age was 42.45 years (SD = 11.32); mean clinical experience, 15.55 years (SD = 9.87). Only five participants (6.25%) reported previous formal training on MUPS communication. The complete demographic and professional characteristics are shown in Table 1.

**Table 1:** Demographic and professional characteristics of the participants (N = 80) Table 1 profiles the 80 clinicians who completed pre‑ and post‑assessments. Age and years licensed are shown as mean ± SD with full range; gender, race, and ethnicity are self‑reported, with Hispanic/Latinx coded as ethnicity. Professional roles—physicians, physician assistants, nurse practitioners, and nurses—differed nonsignificantly on demographics (χ²(3) = 4.22, p = 0.239). Percent totals may vary slightly due to rounding, and only five participants (6.25%) reported prior formal training in MUPS communication. ᵃ Selected > 1 race category (multiracial).

Variable	Category / statistic	n / value	%
Age (years)	Mean	42.45	—
	SD	11.32	—
	Range	24-72	—
Years licensed	Mean	15.55	—
	SD	9.87	—
	Range	0-40	—
Gender (self-identified)	Male	39	48.8
	Female	41	51.3
Race	White / Caucasian	49	61.3
	Multiracial^a^	17	21.3
	Black / African American	7	8.8
	Asian	7	8.8
Ethnicity	Hispanic / Latinx	16	20.0
Previous MUPS training	Formal course completed	5	6.3
Professional role	Physician	23	28.8
	Physician assistant	21	26.3
	Nurse practitioner	13	16.3
	Nurse	23	28.8

Intervention components

The intervention consisted of a single 30-minute psychosomatic education session delivered on-site in three segments to ensure consistency and clarity of content.

Didactic Mini-Lecture

The first segment was a 10-minute didactic mini-lecture introducing foundational biopsychosocial principles [17], reviewing characteristic MUPS presentations (such as chronic pain, fatigue, and gastrointestinal issues), and discussing empathic validation strategies [3,4]. Standardized slides were used to promote internal validity by ensuring all participants received the same core information.

Case-Based Discussion and Role Play

The second segment was a 20-minute case-based discussion and role play. A clinical scenario based on Clarke's approach [23] illustrated mind-body explanations and offered practical avenues for clinicians to explore psychosocial contributors. The participants then engaged in brief role plays that emphasized open-ended questioning, reflective listening, and normalizing stress-related etiologies [1,6]. Facilitated group dialogue addressed adverse childhood experiences (ACEs) and "red flag" psychosocial signs [24]. This structured role-play format enhanced methodological soundness by allowing all attendees to practice and observe standardized communication techniques.

Resource Materials

The final segment consisted of a one‑page resource sheet that distills key “red‑flag” indicators, exemplar ACE‑screening questions [24], and standardized validating phrases for MUPS consultations. Created for rapid, point‑of‑care use, the handout supports consistent language and minimizes implementation variability across clinician roles. Example prompts include, “Have you experienced any significant traumas in childhood that you feel may still affect you?” and “Are there past events or stressors that seem related to your current physical complaints?”

Assessment measures

Measurement Approach

Four instruments were used to capture changes in clinical awareness, knowledge, and comfort regarding MUPS, thereby strengthening statistical validity through multiple data sources. Four external subject-matter experts assessed item relevance (I-CVI = 0.92) and completed think-aloud cognitive interviews, prompting minor wording changes to SSS-8 items 3 and 6. Phase 2: Internal-consistency reliability (Cronbach’s α = 0.71 pre; 0.78 post) and exploratory factor analysis produced a two-factor model (eigenvalues = 3.72 and 2.89; cumulative variance = 56.7 %; loadings ≥ 0.46). Although under the classic 10:1 participant-to-item heuristic, factor loadings and communalities exceeded stability thresholds, supporting provisional structural validity. Each measure was administered pre- and post-training; only the Jefferson Scale of Physician Empathy-Health Professions (JSPE-HP) was administered post-intervention owing to time constraints.

Adapted Somatic Symptom Scale-8 (SSS-8)

In the Adapted Somatic Symptom Scale-8 (SSS-8), originally developed by Gierk and colleagues to measure patient-reported somatic symptoms [25], items were reworded to assess clinicians' awareness of eight common psychosomatic complaints. Each endorsed item received one point (range: 0-8). This adaptation targeted internal validity by aligning the scale with the study's focus on clinician recognition of somatic presentations. Psychometrics from prior validation studies indicate acceptable reliability in patient samples [25], and in this project, internal consistency ranged from α = 0.71 (pre) to α = 0.78 (post). Factor analysis confirmed these items loaded on a distinct "somatic awareness" factor (see Table 2). Complete details about the adaptation, scoring, and psychometric properties are provided in Table 3.

**Table 2:** Exploratory factor analysis summary: eigenvalues and variance Principal axis factoring with Varimax rotation was conducted on 13 items (eight from the adapted SSS-8 and five from PIKQ1) using 160 item-level observations. KMO = 0.78; Bartlett’s χ²(78) = 443.62, p = 0.001. Adapted SSS-8 score range = 0–8; higher values indicate greater clinician recognition of somatic symptoms.

Factor	Eigenvalue	% variance explained	Cumulative % variance
Factor 1	3.72	31.0	31.0
Factor 2	2.89	25.7	56.7

**Table 3:** Adapted Somatic Symptom Scale-8 (SSS-8)

Aspect	Description
Original context	Developed by Gierk et al. [25] to measure patient-reported somatic symptom burden. Original prompt: "During the past seven days, how much have you been bothered by the following problems?" Eight items capturing common somatic complaints rated on a five-point Likert scale (0 = Not at all to 4 = Very much).
Adaptation purpose	Adapted to measure clinicians' awareness of frequent psychosomatic complaints reported by patients with medically unexplained physical symptoms (MUPS) [1-4].
Adapted prompt	"Which of the following symptoms do patients with medically unexplained psychosomatic complaints most frequently report? (Select all that apply)."
Items	1. Stomach or bowel problems 2. Back pain 3. Pain in extremities or joints 4. Headaches 5. Dizziness 6. Chest pain or shortness of breath 7. Feeling tired or having low energy 8. Trouble sleeping
Scoring	• Each selected item = 1 point• Total possible score: 0-8• Higher scores indicate greater recognition/awareness of key somatic presentations.
Psychometric properties	• Original patient version: internal consistency of α ≈ 0.81 [25]. Adapted version: internal consistency α = 0.71 (pre) to α = 0.78 (post). Factor analysis identified a distinct somatic awareness factor (Factor 1).
Notes	Adapted with permission for research/educational use. Further validation recommended for clinician awareness application.

Psychosomatic Illness Knowledge Questionnaire (PIKQ)

The Psychosomatic Illness Knowledge Questionnaire (PIKQ) is a five-item measure that assesses clinicians' knowledge of psychosomatic factors, including psychological triggers, trauma history, and functional versus organic etiologies [17,22]. Participants selected statements they believed were accurate about MUPS; one point was awarded for each correct selection, yielding scores of up to 5. While its primary support is face validity, factor analysis corroborates the existence of a distinct "psychosomatic knowledge" construct, bolstering internal consistency (see Table 2) [17,18,22]. The PIKQ structure, specific statements, and validation information are detailed in Table 4.

**Table 4:** Psychosomatic Illness Knowledge Questionnaire (PIKQ)

Aspect	Description
Purpose	Assess knowledge of key psychosomatic illness features. Addresses common challenges in differentiating psychogenic from organic etiologies [2,17]
Structure	Respondents select all statements they believe accurately characterize MUPS or psychosomatic illness.
Items	1. "Psychosocial factors strongly affect MUPS presentations." 2. "Early trauma (e.g., ACEs) can underlie chronic somatic issues." 3. "MUPS frequently co-occur with stress or anxiety." 4. "MUPS management often includes validation & psychosocial care." 5. "Recognizing functional vs. organic etiologies is essential."
PIKQ Scoring	Each correctly identified true statement = 1 point. Possible range: 0-5. Higher scores indicate stronger psychosomatic knowledge
Validity	Face validity grounded in MUPS literature [2,17,18]. Factor analysis confirmed a distinct psychosomatic knowledge domain (Factor 2; Table 2).
Notes	Newly developed for research/educational purposes. Further psychometric testing is encouraged.

Knowledge and Comfort in Treating MUPS Questionnaire (KCTMQ)

The Knowledge and Comfort in Treating MUPS Questionnaire (KCTMQ) was composed of two single Likert-scale items. KCTMQ1 measured self-reported knowledge of MUPS, and KCTMQ2 measured comfort in caring for MUPS, both ranging from 1 (not at all) to 5 (very). This parsimonious design addressed common clinical uncertainties observed in MUPS management [6,17]. Each item's isolated measure of a core dimension (knowledge or comfort) contributed to the study's overall methodological soundness, given that self-efficacy perceptions often predict clinicians' engagement with new skills. Full descriptions of these items and their scoring approach are available in Table 5.

**Table 5:** Knowledge and Comfort in Treating MUPS Questionnaire (KCTMQ)

Aspect	Description
Overview	Designed to capture perceived knowledge and comfort in managing MUPS. Addresses research showing low perceived knowledge and comfort contribute to clinicians' difficulties [6,17]
Items	KCTMQ1: "How knowledgeable do you feel about identifying, evaluating, and managing patients with medically unexplained psychosomatic symptoms?" KCTMQ2: "How comfortable do you feel providing ongoing care and support for patients with medically unexplained psychosomatic symptoms?"
Scale	Five-point Likert scale: 1 = not at all, 5 = very
Scoring	Each item was analyzed separately. Total possible range = 1-5 per item. Higher scores = greater perceived knowledge (KCTMQ1) or comfort (KCTMQ2)
Notes	Single-item format lacks extensive psychometric validation (see questionnaire-development guidance [26]). Interpreted as self-reported indicators. Effect sizes used to assess pre-post changes.

Jefferson Scale of Physician Empathy (JSPE-HP)

Lastly, the Health Professions Version of the Jefferson Scale of Physician Empathy (JSPE-HP) was administered only post-intervention to examine correlations with psychosomatic knowledge and somatic awareness [27,28]. This 20-item instrument uses a seven-point Likert scale (1 = strongly disagree, 7 = strongly agree), with higher scores denoting stronger empathy. Internal consistency was α = 0.84, which is consistent with the typical psychometric performance of this measure in health professions contexts [19,20]. The structure, domains, and scoring information for the JSPE-HP are provided in Table 6. 

**Table 6:** Jefferson Scale of Physician Empathy-Health Professions (JSPE-HP) The complete JSPE-HP items and scoring details cannot be displayed here due to copyright restrictions. The full instrument is available on request from the copyright holder.

Aspect	Description
Purpose	Measures empathy in healthcare providers. Included post-intervention to explore correlations between empathy levels and gains in psychosomatic communication skills [19,20].
Structure	20 items rated on a seven-point Likert scale (1 = strongly disagree, 7 = strongly agree). Addresses three domains: (1) perspective taking, (2) compassionate care, and (3) standing in the patient's shoes.
Scoring	Total scores range from 20 to 140. Higher scores reflect greater empathic disposition [19].
Psychometric Properties	Typical reliability is α = 0.80-0.90 [19,20]; α = 0.84 in this sample.
Notes	Used to assess correlation with psychosomatic knowledge and comfort scores. No causal inferences about empathy changes are made. The JSE was used in this study with permission from Thomas Jefferson University.

Measurement Selection Considerations and Psychometric Limitations

Given the specialized focus on MUPS communication and the quality improvement framework of this study, we encountered several constraints in measurement selection that warrant explanation. While established measures for assessing clinical knowledge and communication in standard medical domains are abundant, few validated instruments specifically address clinician competencies in psychosomatic communication. This measurement gap reflects the broader educational deficit in MUPS training identified in our literature review.

The decision to adapt existing measures (SSS-8) and develop new instruments (PIKQ, KCTMQ) was guided by three practical considerations. First, the educational focus of our intervention required assessment tools aligned with our specific learning objectives regarding psychosomatic awareness and biopsychosocial integration. Second, participant availability constraints in the community health setting necessitated brief, efficient measures that could be completed within time-limited educational sessions. Third, as a quality improvement initiative rather than a definitive efficacy trial, we prioritized feasibility and educational relevance over comprehensive psychometric validation.

We acknowledge measurement validity limitations and took several steps to mitigate these concerns. Initial informal pilot testing with four clinicians not included in the final sample provided feedback on item clarity and face validity. The factor analysis demonstrating distinct loadings for somatic awareness and psychosomatic knowledge constructs provided preliminary structural validation. Additionally, we employed methodological triangulation by collecting complementary qualitative data to corroborate quantitative findings, enhancing interpretive validity despite measurement limitations.

While acknowledging these instruments require further validation, they provided actionable insights into intervention effects within the constraints of our quality improvement framework. Future research should build on these preliminary instruments through more extensive psychometric validation in diverse clinical samples.

Data collection and analysis

Data Collection Procedures

All measures (except the JSPE-HP) were administered immediately before and after the training session. To maintain internal validity, participants were assigned unique numeric codes and completed electronic surveys under uniform conditions. This approach minimized the risk of misidentifying responses and preserved anonymity.

Scoring Methodology

Adapted SSS-8 and PIKQ responses were coded as "selected/not selected" for each item, and summed scores reflected either total awareness (SSS-8) or knowledge (PIKQ). KCTMQ items were scored on a 1-to-5 scale, with higher values indicating greater knowledge or comfort. Fewer than 2% of data points were missing; these were handled by listwise deletion to retain methodological soundness without introducing imputation bias.

Factor Analysis

Principal axis factoring with Varimax rotation was performed on combined pre-/post-intervention item-level responses (N = 160) for SSS-8 and PIKQ items. As summarized in Table 2, principal-axis factoring (Varimax) on combined SSS-8 + PIKQ items (N = 160) produced a two-factor solution (eigen = 3.72 and 2.89; cumulative variance = 56.7%; KMO = 0.78; Bartlett’s χ²(78) = 443.6, p < .001). This analysis supported the separation of somatic awareness (SSS-8 items) and psychosomatic knowledge (PIKQ items). The factor structure corroborated the study's conceptual basis by indicating that clinicians' awareness of somatic presentations and knowledge of psychosomatic influences are related yet distinct constructs, reinforcing internal validity. 

Statistical Analysis

Following reliability guidelines for study scales (Cronbach’s α ≥ 0.70) [29] and questionnaire pre-test heuristics showing that four to five interviews detect over 80 % of usability problems [30], Shapiro-Wilk tests indicated non-normality in SSS-8 and PIKQ scores; therefore, Wilcoxon signed-rank tests were applied [31]. KCTMQ items conformed to normality assumptions and were analysed with paired-samples t-tests. Holm’s sequentially rejective procedure controlled the family-wise error rate across multiple comparisons [32]. Effect sizes (Cohen’s d for t-tests; rank-biserial correlations for Wilcoxon) quantified practical significance [33]. All inferential tests used a two-tailed significance level of α = 0.05. In parallel, weekly referral counts were plotted on a Shewhart X̄ control chart with ±3-sigma limits (α ≈ 0.0027 per point) to provide an additional check on false-positive findings.

Secondary Analyses

Secondary analyses examined role-specific differences among physicians, physician assistants, nurse practitioners, and nurses using Kruskal-Wallis or ANOVA, as appropriate. Correlations between JSPE-HP scores and changes in MUPS knowledge or somatic awareness explored links between empathy and uptake of psychosomatic skills [27,28,34].

Qualitative Assessment

To complement the quantitative data, open-ended reflections were collected post-intervention and thematically analyzed following Braun and Clarke's reflexive approach [35,36]. Two independent coders assigned preliminary codes, which were then reviewed to resolve discrepancies by consensus (κ = 0.822). This systematic procedure aimed to capture the breadth of participants' subjective experiences, thereby enhancing methodological rigor through triangulation of quantitative and qualitative results [35,36].

Ethical considerations

The project was reviewed by the Arizona State University Institutional Review Board (IRB ID STUDY00021486), which confirmed that no additional oversight was required. Participation was voluntary and anonymous, with informed consent obtained prior to data collection.

## Results

Participant characteristics and engagement

Of the 346 clinicians invited, 81 (23.4%) participated, and 80 (23.1%) completed both pre- and post-assessments (1.2% attrition). This high completion rate of 80 out of 81 participants (98.8%) suggests the feasibility of incorporating brief psychosomatic communication training into time-constrained healthcare education settings. The final sample of 80 participants comprised 23 physicians (28.8%), 21 physician assistants (26.3%), 13 nurse practitioners (16.3%), and 23 nurses (28.8%), with 41 (51.3%) identifying as female. The mean clinical experience was 15.55 years (SD = 9.87).

Changes in communication knowledge and skills

**Figure 2:**
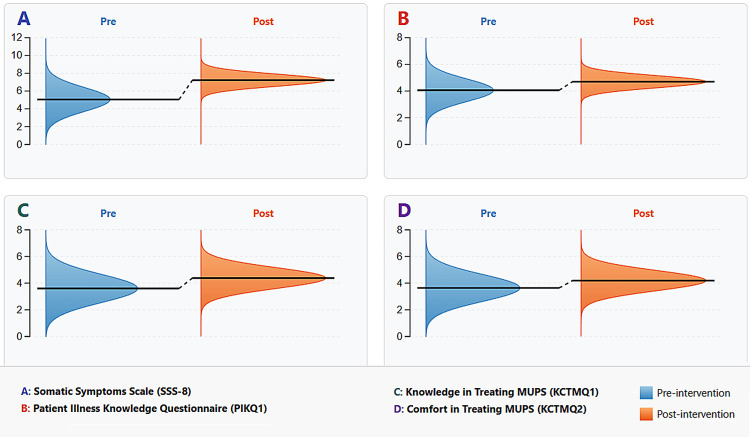
Pre- and post-intervention score distributions by measure Horizontal violin plots compare pre‐ (blue) and post‐ (red) distributions for four outcome measures (A: adapted SSS‐8, B: PIKQ1, C: KCTMQ1, D: KCTMQ2). The dashed reference line marks the mean, and the spread indicates score variability.

All four primary measures showed significant improvement following the brief communication training (Table 7, Figure 2). Wilcoxon's signed-rank tests and paired t-tests revealed substantial gains (p < 0.001) in somatic-symptom recognition (adapted SSS-8), psychosomatic knowledge (PIKQ), and self-reported knowledge and comfort (KCTMQ1, KCTMQ2). Clinicians demonstrated markedly improved recognition of common patient-reported symptoms, with a large effect size (d = 2.04), and increased understanding of psychosomatic principles (d = 0.94). Self-reported communication competence and comfort also showed medium-to-large effect sizes (d = 0.88 and d = 0.79, respectively). Nonparametric tests confirmed these results, and Holm-Bonferroni adjustments indicated all findings remained significant [33].

**Table 7:** Pre–post intervention outcome measures (N = 80) Paired-samples t-tests were conducted for these comparisons. All were significant at p = 0.001 (after Holm’s correction). Confidence intervals (CIs) are provided in brackets.

Outcome measure	Pre-intervention (M [SD])	Post-intervention (M [SD])	Mean change (Δ) [95% CI]	t(df)	p-value	Cohen’s d
Adapted SSS-8 (range: 0–8)	5.05 (1.34)	7.22 (0.69)	+2.17 [1.88, 2.47]	13.97 (79)	0.001	2.04
PIKQ1 (range: 0–5)	4.06 (0.85)	4.70 (0.46)	+0.64 [0.45, 0.83]	9.17 (79)	0.001	0.94
KCTMQ1 – Knowledge in Treating MUPS (range: 1–5)	3.60 (1.06)	4.38 (0.78)	+0.77 [0.54, 1.01]	8.92 (79)	0.001	0.88
KCTMQ2 – Comfort in Treating MUPS (range: 1–5)	3.64 (1.01)	4.19 (0.76)	+0.55 [0.36, 0.74]	7.80 (79)	0.001	0.79

Role‐specific analysis and perspectives

Analysis by professional role revealed noteworthy variations in comfort and knowledge scores. Although all groups showed significant improvement overall (time × role, p = 0.02), the magnitude of gains differed. As presented in Table 8, nurses, who began with a mean SSS-8 score of 4.41 ± 0.97, showed the largest absolute gain (Δ = +2.59 points, post 7.00 ± 0.52 on the 0-8 scale) in recognizing patient-reported symptoms. Physician assistants recorded robust increases in comfort when discussing psychosocial factors. Nurse practitioners also improved from baseline but showed the smallest absolute gain in comfort (KCTMQ2; post-score 4.15 ± 0.58 on a 1-5 scale), indicating potential barriers that may be more pronounced in their workflow or scope of practice. A forthcoming manuscript will expand on baseline-to-post-training shifts for each role, shedding light on how different disciplines respond to psychosomatic-communication training and illustrating discipline-specific barriers or facilitators in managing medically unexplained physical symptoms [6].

**Table 8:** Post-intervention outcome means by professional role (N = 80) Omnibus Kruskal–Wallis tests indicated significant group differences in post-intervention scores for all measures: adapted SSS-8, H(3) = 10.60, p = 0.014. KCTMQ1, H(3) = 32.20, p = 0.001. KCTMQ2, H(3) = 16.67, p = 0.001. PIKQ1, H(3) = 18.55, p = 0.001.

Professional role	n	SSS-8(M±SD)	SE	KCTMQ1(M±SD)	SE	KCTMQ2(M±SD)	SE	PIKQ1(M±SD)	SE
Physicians	23	7.52 ± 0.59	0.12	4.78 ± 0.42	0.09	4.17 ± 0.63	0.13	4.91 ± 0.29	0.06
Physician assistants	21	7.00 ± 0.84	0.18	4.62 ± 0.56	0.12	4.52 ± 0.67	0.15	4.71 ± 0.33	0.07
Nurse practitioners	13	7.46 ± 0.66	0.18	4.31 ± 0.75	0.21	4.15 ± 0.58	0.16	4.23 ± 0.44	0.12
Nurses	23	7.00 ± 0.52	0.11	3.78 ± 0.48	0.10	3.91 ± 0.54	0.11	4.74 ± 0.36	0.08

Nonparametric and role-specific analyses

To verify our parametric findings, we conducted Wilcoxon's signed-rank tests for each outcome measure. As presented in Table 9, these nonparametric results confirmed statistically significant improvements from pre- to post-intervention across all four measures (p = 0.001). Specifically, the Adapted SSS-8 showed a large rank improvement (Wilcoxon's W = 28.0, r ≈ 0.86), and both KCTMQ1 (Knowledge) and KCTMQ2 (Comfort) demonstrated large effect sizes as well (W = 319.5, r = 0.76; W = 318.0, r ≈ 0.73, respectively). For PIKQ1, nearly all participants (74 out of 80) improved from baseline to post-training, underscoring the uniformity of gains in psychosomatic knowledge; because of this near-universal improvement, a rank-biserial correlation was not computed. Taken together, these nonparametric outcomes reinforce the robustness of our parametric results, indicating consistent and substantial increases in somatic symptom awareness, psychosomatic knowledge, and clinician confidence in treating MUPS.

**Table 9:** Nonparametric outcome confirmations (Wilcoxon's signed-rank tests) The PIKQ1 effect size was not computed because nearly all participants improved (74/80, 92.5%). All Wilcoxon's signed-rank tests confirmed significant pre–post improvements (p = 0.001).

Outcome measure	Wilcoxon's W	p-value	Effect size (r)
Adapted SSS-8	28.0	0.001	≈0.86
PIKQ1	0.0	0.001	Not computed
KCTMQ1 (Knowledge in Treating MUPS)	319.5	0.001	0.76
KCTMQ2 (Comfort in Treating MUPS)	318.0	0.001	≈0.73

Qualitative insights on communication practice changes

**Figure 3:**
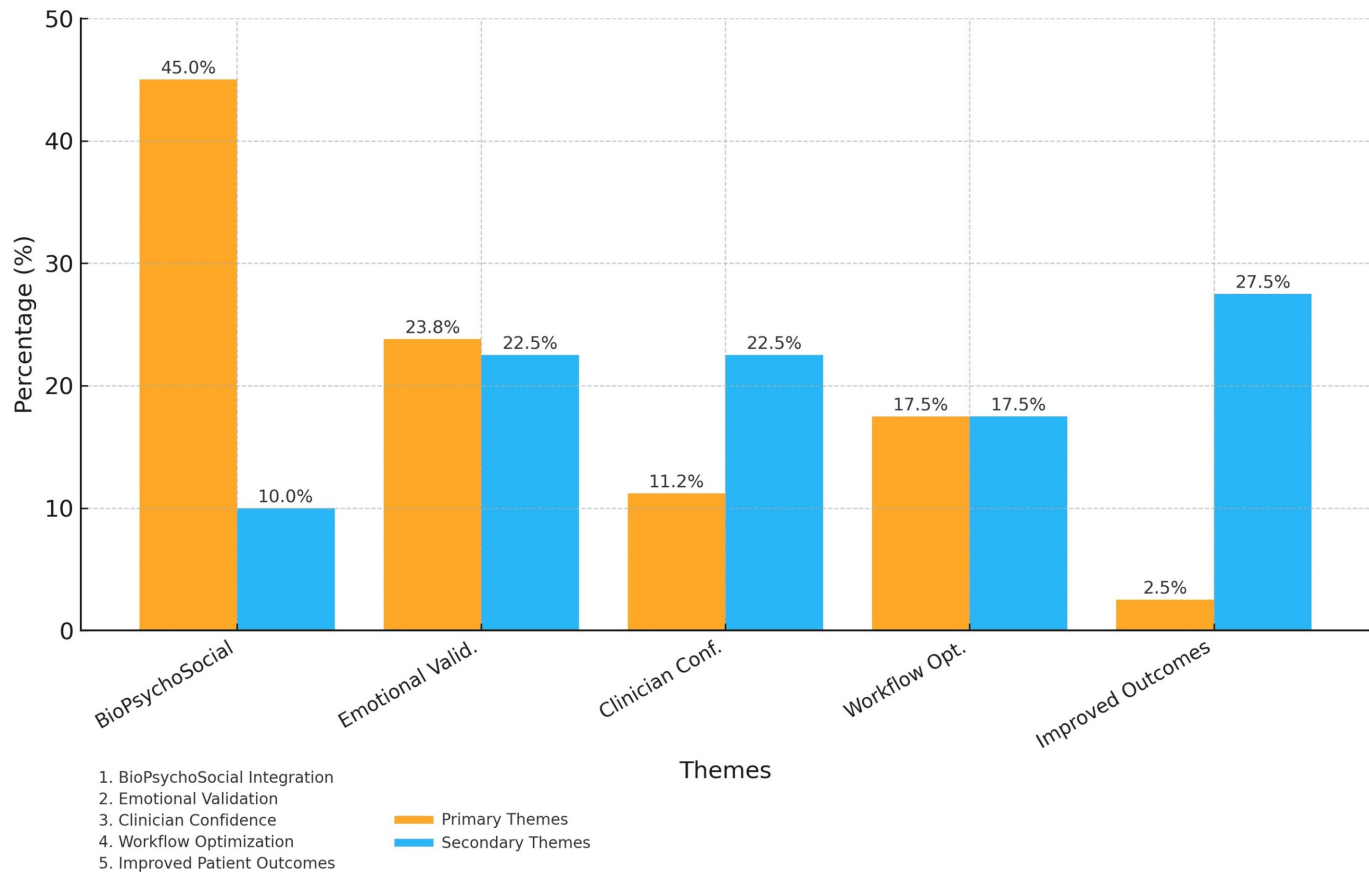
Qualitative themes identified from MUPS education module feedback This stacked bar chart summarizes the thematic analysis of 78 post-training reflections. The y-axis shows the percentage of comments containing each theme. Orange (upper) segments represent primary mentions; blue (lower) segments represent secondary mentions. The five themes—biopsychosocial integration, emotional validation, clinician confidence, workflow optimization, and anticipated patient outcomes—reflect clinicians’ intended practice changes after training.

The analysis of open-ended reflections (n = 78, 97.5% response rate) revealed five themes representing intended changes in clinical communication practices [35,36]. Figure 3, which illustrates theme distribution, shows that clinicians emphasized different aspects of communication improvement. As shown in Table 10, many clinicians emphasized incorporating psychosocial factors earlier in consultations, expressing intentions to validate patient experiences more thoroughly. Others cited increased confidence in initiating potentially sensitive discussions or planned to optimize communication workflows by screening for psychosocial stressors at the outset. Some participants anticipated improved patient outcomes through better mind-body explanations, while a small subset (n = 2, 2.5%) questioned the practical value of deeper psychosomatic inquiry.

**Table 10:** Qualitative themes from open-ended reflections on communication practice changes (N = 78) Five numbered themes (1–5) were derived via reflexive thematic analysis of 78 clinician reflections. Percentages show the proportion of total coded responses; “primary” indicates the dominant theme in a comment, while “secondary” marks an additional but less prominent theme. Themes: 1 = Biopsychosocial integration; 2 = Emotional validation techniques; 3 = Clinician confidence; 4 = Communication-workflow optimization; 5 = Anticipated patient-outcome improvements.

Theme	Primary n (%)	Secondary n (%)	Representative quotation
Theme 1: Biopsychosocial integration	35 (45.0 %)	8 (10.0 %)	“I hope to bring up stress sooner, knowing this could help prevent unnecessary follow-ups.”
Theme 2: Emotional validation techniques	19 (23.8 %)	18 (22.5 %)	“I want to be more patient when the cause may not be purely physical.”
Theme 3: Clinician confidence	9 (11.2 %)	18 (22.5 %)	“This session gave me more confidence in validating patients’ experiences rather than just ruling out causes.”
Theme 4: Communication workflow optimization	14 (17.5 %)	14 (17.5 %)	“Asking about trauma or stress at the start may help avoid needless labs and imaging.”
Theme 5: Anticipated patient outcome improvements	2 (2.5 %)	21 (27.5 %)	“By connecting mind and body, I think I can catch issues sooner. I want to reduce the cycle of frustration.”

Relationship between knowledge and empathy

The mean Jefferson Scale of Physician Empathy score (JSPE-HP) was 118.46 (SD = 10.84), showing modest positive associations with psychosomatic knowledge (r ≈ 0.35, p = 0.04) and comfort discussing psychosocial factors (r ≈ 0.41, p = 0.04). Factor analysis confirmed that somatic awareness and psychosomatic knowledge are distinct but related domains. Mediation analysis indicated that knowledge did not fully explain how somatic awareness translates into communication confidence, suggesting additional moderating factors [26].

## Discussion

Summary of key findings

Challenging conventional assumptions that effective MUPS communication training requires extensive curricular intervention [6], this study demonstrated significant clinical improvements using a concise, 30-minute psychosomatic education module. Clinicians showed substantial enhancements in their capacity to communicate with patients presenting medically unexplained symptoms, with effect sizes ranging from medium (d = 0.79 for comfort) to large (d = 2.04 for symptom recognition) across all outcome measures. Most notably, pronounced improvements were seen in recognizing somatic complaints and psychosomatic knowledge, accompanied by significant gains in self-reported communication competence. Complementing these quantitative findings, qualitative reflections revealed participants’ intentions to implement specific communication strategies, particularly earlier psychosocial screening and empathic validation techniques, further supporting the efficacy of targeted interventions in strengthening clinicians’ readiness to discuss unexplained symptoms within a patient-centered framework [1-5].

Clinician communication skills

Anchored in Engel’s biopsychosocial model [10] and adult learning theory [12], our findings reinforce that skillful validation of somatic symptoms and the exploration of psychosocial contributors may help reduce patient dissatisfaction and clinician frustration, bridging common communication gaps in MUPS care [3,4,19]. Participants emphasized the importance of acknowledging patients’ subjective experiences before introducing potential psychological or social factors, echoing Clarke’s mind-body explanations [11]. By incorporating open-ended questions and empathic statements, clinicians can align more closely with best practices in patient-centered care [1,6,17].

Educational strategies

The brief intervention design followed adult learning principles, combining a concise mini-lecture, case-based discussion, and role-play in a single 30-minute session [12]. This approach likely contributed to the substantial gains in knowledge and comfort, as participants could immediately practice the communication skills taught. Similar short-format interventions could be integrated into busy clinical education environments without requiring major curricular overhauls [1,5]. By focusing specifically on communication rather than diagnostic algorithms, this training fills an educational gap often overlooked in standard curricula [3,4,19].

Comparison with prior literature

Recent reviews demonstrate that undergraduate programs still deliver only sporadic, culturally limited teaching on medically unexplained or persistent physical symptoms [5,9]. Most published MUPS-communication courses entail six to 14 hours. A 14-hour postgraduate course improved reassurance skills but required protected time [6]. Similarly, a two-session Individual Challenge Inventory Tool cluster-RCT still demanded multiple encounters [37]. Achieving comparable or larger gains with a single 30-minute micro-learning session therefore offers a favorable effort-to-benefit ratio that compares well with multi-session MUPS programs (d = 0.79-2.04). To our knowledge, this is the first cross-disciplinary study showing that a sub-one-hour intervention can match or exceed effect sizes of far longer programs without requiring release time from clinical duties.

Qualitative insights

Qualitative reflections showed that many clinicians intend to integrate psychosocial considerations earlier in patient visits, optimize clinical workflows by screening for adverse childhood experiences, and offer validating language that normalizes the mind-body connection. Although these reflections signal promising shifts, the remarks represent intended or anticipated behavior changes rather than confirmed clinical practices [24,25]. Future longitudinal or observational studies could assess whether these strategies are adopted consistently over time and whether they lead to measurable improvements in patient outcomes [1,4,19].

Study limitations

Non-Response Bias and Hawthorne Effect

Only 23% of the invitees (80/346) completed both surveys, leaving 266/346 non-responders (76.9%). The high pre-session loss, driven largely by unanswered e-mails and time pressures, means that respondents may be disproportionately motivated to improve MUPS communication. This selective participation could inflate observed effect sizes and temper generalizability. In addition, a Hawthorne effect cannot be excluded; clinicians may have altered their behavior simply because they knew they were being observed. Third, reliance on self-reported outcomes introduces social-desirability bias, potentially inflating perceived communication improvements.

Immediate Post-Test Timing

All outcomes were measured immediately post-session, which precludes conclusions about skill decay. Systematic reviews indicate that communication and empathy-training effects can remain detectable for at least six months, albeit at reduced magnitude [38-40]. Comparable decay patterns have been demonstrated in other clinical competencies, where spaced refreshers improve retention [41]. Evidence in patients with medically unexplained symptoms likewise shows sustained or even enhanced effects at six to 12 months when multicomponent or booster formats are used [42]. Future evaluations of this module should therefore include follow-up assessments and low-dose reinforcement to test durability in routine practice.

Uncontrolled Design and Type I Error

The single-group, pre-post design is susceptible to Type I error-detecting improvements driven by secular trends rather than the module itself [43]. To curb this risk, we limited analyses to prespecified outcomes, reported effect sizes with ninety-five-percent confidence intervals, and interpreted quantitative changes only when corroborated by convergent qualitative themes, consistent with mixed-methods-triangulation guidance [44,45]. Statistical-process-control concepts further help distinguish common-cause variation from special-cause signals [46-49].

*Profession-Specific Powe*r

Although baseline demographics did not differ significantly by role, the profession-specific cells (n = 13-23) are underpowered for definitive subgroup inference; role-level findings should therefore be considered exploratory. 

Measurement and methodological considerations

Because the study employed a single-group, pre-post design, improvements cannot be conclusively attributed solely to the intervention [8,9]. The Adapted Somatic Symptom Scale-8 and the single-item KCTMQ scales provided efficient pre-post assessments of MUPS awareness, knowledge, and comfort, but they warrant further validation in larger or more diverse clinician samples [16,18]. Longitudinal assessments and observational measures (for example, standardized-patient encounters) would provide stronger evidence of sustained behavior change and clarify how newly gained communication competencies translate to real-world settings [3,4,19].

The pragmatic measurement approach in this quality-improvement study balanced educational relevance with psychometric rigor, selecting instruments that directly assessed our specific learning objectives, despite limited prior validation. The convergence between quantitative improvements and qualitative themes offers supporting evidence for intervention effects that go beyond what any single measurement approach can provide. Nevertheless, because time and resource constraints precluded expert-panel content validity indexing, cognitive interviewing with saturation, or test-retest analysis, the current evidence base is limited to face validity, internal consistency, and an exploratory factor solution. A multi-site study is underway to complete COSMIN-recommended validity steps-including content validity, temporal stability, and hypothesis-testing correlations [50,51].

Implications for clinical practice

Short-format psychosomatic communication training can feasibly address critical educational gaps in settings where MUPS are highly prevalent. Embedding this module in undergraduate medical education, interprofessional team development, and continuing professional development offerings could better equip clinicians to handle the biopsychosocial complexity of unexplained symptoms [5,11]. By tailoring the module to different professional roles, acknowledging varied baseline knowledge and comfort levels, healthcare systems may foster cohesive, team-based approaches that reduce unnecessary testing and improve patient satisfaction [14,27,28].

Future directions

While immediate post-training improvements were considerable, subsequent research should focus on the durability of these gains and their impact on patient-centered metrics such as satisfaction, decreased healthcare utilization for MUPS, or improved symptom outcomes [6,7]. Randomized controlled trials using standardized patients or direct observation would yield robust data on whether clinicians consistently employ these communication techniques in everyday practice. Additional work could examine how to tailor short-format psychosomatic modules to diverse cultural contexts, as mind-body conceptions and symptom reporting can vary significantly among different patient populations [14,19,28]. Finally, digital “refresher” modules and periodic feedback sessions could help sustain and reinforce the communication skills acquired [29].

## Conclusions

This study reveals a powerful opportunity in healthcare education, i.e., just 30 minutes of targeted psychosomatic training, can transform how clinicians approach the persistent challenge of medically unexplained symptoms. The substantial improvements in clinicians' capacity to recognize somatic complaints, validate patient experiences, and integrate psychosocial dimensions represent more than academic gains; they address a fundamental communication gap that frustrates both patients and providers daily. While longer-term follow-up remains necessary, these findings challenge the assumption that addressing complex clinical communication requires an extensive curricular overhaul. Instead, by strategically equipping clinicians with patient-centered explanation models, validation techniques, and psychosocial screening approaches, healthcare systems can implement practical, time-efficient solutions that fit within existing educational frameworks. Beyond immediate clinical benefits, this approach holds promise for reducing unnecessary testing, improving patient satisfaction, and ultimately transforming the therapeutic relationship in MUPS care, demonstrating that sometimes the most profound changes in clinical practice begin with just 30 minutes of focused attention.
